# Observed drought indices show increasing divergence across Europe

**DOI:** 10.1038/s41598-017-14283-2

**Published:** 2017-10-25

**Authors:** James H. Stagge, Daniel G. Kingston, Lena M. Tallaksen, David M. Hannah

**Affiliations:** 1 University of Oslo, Department of Geosciences, Oslo, Norway; 2 University of Otago, Department of Geography, Dunedin, New Zealand; 3 University of Birmingham, Department of Geography, Earth and Environmental Sciences, Birmingham, United Kingdom; 4 Present Address: Utah State University, Department of Civil and Environmental Engineering, Logan, UT USA

## Abstract

Recent severe European droughts raise the vital question: are we already experiencing measurable changes in drought likelihood that agree with climate change projections? The plethora of drought definitions compounds this question, requiring instead that we ask: how have various types of drought changed, how do these changes compare with climate projections, and what are the causes of observed differences? To our knowledge, this study is the first to reveal a regional divergence in drought likelihood as measured by the two most prominent meteorological drought indices: the Standardized Precipitation Index (SPI) and the Standardized Precipitation-Evapotranspiration Index (SPEI) across Europe over the period 1958–2014. This divergence is driven primarily by an increase in temperature from 1970–2014, which in turn increased reference evapotranspiration (ET_0_) and thereby drought area measured by the SPEI. For both indices, Europe-wide analysis shows increasing drought frequencies in southern Europe and decreasing frequencies in northern Europe. Notably, increases in temperature and ET_0_ have enhanced droughts in southern Europe while counteracting increased precipitation in northern Europe. This is consistent with projections under climate change, indicating that climate change impacts on European drought may already be observable and highlighting the potential for discrepancies among standardized drought indices in a non-stationary climate.

## Introduction

The IPCC report on extreme events and disasters^1^ cites a greater uncertainty in capturing recent drought trends compared to other natural hazards. At the global scale, studies have shown either increases or negligible changes in meteorological drought^2–4^. Southern Europe is considered a hot-spot for drought change under climate change^5–9^. While some pan-European studies have identified a slight increase in drought area for the continent^10^, continental scale trend studies have partially been confounded by the distinct north-south dipole that yields decreased drought frequency in northern Europe and an increase for southern Europe^6,11,12^. Therefore, results are contingent on the geographical domain, which can shift spatially-averaged values for ‘Europe’. Another major source of uncertainty is due to differences in drought index formulation, data sources, and selected time period^3,13–16^.

The Standardized Precipitation Index (SPI^17,18^) and (SPEI) Standardized Precipitation-Evapotranspiration Index^19^ normalize precipitation and climatic water balance (precipitation minus ET_0_), respectively, accumulated over a given number of months. The SPI has become widely used because of its low data requirements, ease of statistical interpretation, and recommendation by the WMO^20,21^. However, the SPI is based solely on precipitation, and thus ignores the role of evaporative loss in the terrestrial water balance. In recognition of this limitation, the SPEI was developed as a complementary drought index, to provide a more complete measure of the climate inputs and losses related to drought. The incorporation of ET_0_, and by implication temperature, in the SPEI calculation has been hypothesized to capture better the projected and observed effects of climate change^19,22,23^; however, this hypothesis has yet to be tested at the continental scale.

Given this research context, we test herein the hypothesis that two closely related drought indices tracked by most drought warning systems, the SPI and SPEI at 6-month resolution (henceforth SPI6 and SPEI6), produce different drought trends across Europe during the recent past. This work builds on prior studies of observed drought and climate trends in Europe^2–4,10,22,24^, providing greater detail and a specific analysis of spatial and temporal divergence between two drought indices in Europe during the last 60 years (1958–2014). Notably, we identify the timing of an increasing deviation between the indices from the late 1980s onwards. We seek to understand the core causes of this deviation by mapping spatial patterns of change, identifying the critical climate variables driving change, and verifying the robustness of findings to methods of calculating ET_0_ (used in the SPEI6). We conclude that observed drought trends in Europe are driven by the north-south dipole in precipitation, superimposed on a Europe-wide increasing trend in reference evapotranspiration, driven by increasing temperatures. These broad trends mirror projections of future drought, providing clear evidence that climate change is already affecting European drought frequency, while also raising a warning that related standardized indices may diverge in a non-stationary climate.

## Results

### Continental Drought Trends

**Figure 1:**
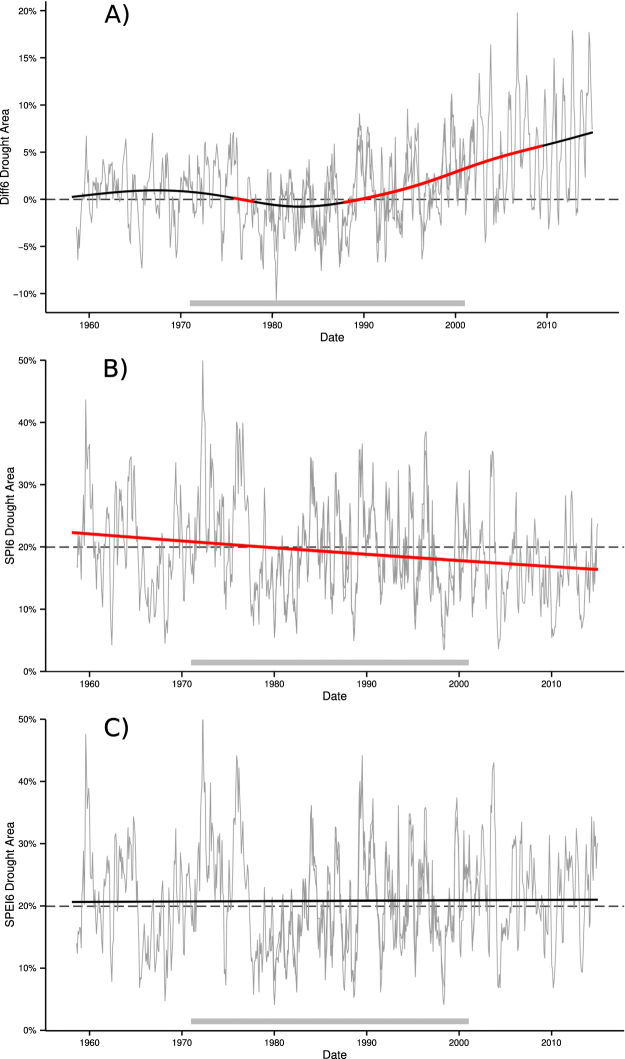
Difference in percent area in drought *A*_*Diff*6_ (**A**), followed by the percent area in drought calculated by (**B**) SPI6, *A*_*SPI*6_, and (**C**) SPEI6, *A*_*SPEI*6_. Historical values for both the WFD and WFDEI are shown in grey, while the fitted common trend in shown in black. Statistically significant trends are shown in red. A grey bar at the bottom shows the reference period.

Observed trends in percent European drought area, i.e. the area fraction of grid cells in drought, were calculated based on the SPI6, SPEI6, along with the difference in percent drought area, *A*_*DIFF*6_ (*A*_*SPEI*6_ minus *A*_*SPI*6_). For this study, drought was defined as SPI6 or SPEI6 below the 20^th^ percentile for each grid cell separately. Long-term trends for these three variables were modeled by non-linear regression using cubic splines along with variables that controlled for recurrent seasonal cycles, temporal autocorrelation, and bias between the two source data sets: the Watch Forcing Data^25^ (WFD, 1958–2001) and the Watch Forcing Data Era-Interim^26^ (WFDEI, 1979–2014). These datasets are based on the well-reviewed ERA-40^27^ and ERA-Interim^28^ gridded climate data, respectively, but have undergone spatial interpolation to improve the resolution to 0.5 × 0.5°, and been subject to additional validation against observed climate records and bias correction based on rain gauge data^26^. Using overlapping datasets with a bias correction intercept increases trend confidence during the common time period (1979–2001) and permits calculation of a common trend spanning the full period (1958–2014). Figure 1 shows *A*_*SPI*6_, *A*_*SPEI*6_, and *A*_*DIFF*6_ for both the WFD and WFDEI after accounting for bias, confirming good agreement between the datasets.

The difference between European drought area measured by SPEI and SPI, *A*_*DIFF*6_, shows a statistically significant trend beginning in the late 1980s that continued until 2014 (Fig. 1A). The trend became non-significant towards the end of the period due to increased uncertainty related to its estimation near the end of the record rather than any marked change in slope. The spline trend indicates a difference in drought area measured by the two indices of −0.4% in 1987, which rose to a maximum of 7.1% in 2014, implying that an additional 7.1% of the European land mass would be considered in drought, if drought were defined by climatic water balance (SPEI) rather than precipitation (SPI) alone. This increase was continuous, with an average increase of 2.8% per decade, and shows no signs of slowing or changing direction. The maximum observed, unsmoothed value of *A*_*DIFF*6_ occurred in September of 2006 (19.8%), when *A*_*SPI*6_ was near the expected mean of 20% based on the drought definition threshold, whereas *A*_*SPEI*6_ showed a relatively severe drought, encompassing 33.5% of Europe. A similar *A*_*DIFF*6_ trend and onset was observed when the E-Obs dataset^29^ was processed by the authors using the same method, lending further confidence to this finding.

Prior to the start of the near-linear increase during the late 1980s, the two drought indices were closely related, with most differences in drought area attributable to noise superimposed onto minor decadal patterns. The monthly difference between *A*_*SPEI*6_ and *A*_*SPI*6_ alternated between a slightly positive period (1958–1976, ${\overline{A}}_{DIFF6}=+\mathrm{1.00 \% }$) and a slightly negative period (1976–1985, (${\overline{A}}_{DIFF6}=-\mathrm{1.33 \% }$). The shift from positive (*A*_*SPI*6_ > *A*_*SPEI*6_) to negative (*A*_*SPI*6_ < *A*_*SPEI*6_) occurred rapidly following the longest drought in the record (1975–1976, Fig. 1A). Representation of this step-change by the spline method was as a short, but statistically significant decreasing trend (Fig. 1A). Ultimately, the trends prior to the onset of significant deviation in the late 1980s were minor, indicating that differences between the indices were stable and randomly distributed, especially when compared to the more severe and continuous trend during the past three decades.

To understand the cause of increasing differences in drought area, we considered the trends in *A*_*SPI*6_ and *A*_*SPEI*6_ separately. These trends clearly show that the observed deviation was mainly caused by significant decreases in SPI-based drought area (*A*_*SPI*6_) (Fig. 1B). The smoothed trend of *A*_*SPI*6_ decreased from 22.3% in Jan 1958 to 16.4% in Dec 2014, or −1.04% per decade. Unlike precipitation-based drought area (*A*_*SPI*6_), water balance drought area (SPEI) did not change significantly during this period, increasing only slightly from 20.6% to 21.1% (Fig. 1C).

### Spatial Drought Trends

Analysis of continental drought trends show that the total area experiencing SPI6 drought in Europe has decreased significantly during the past 56 years (Fig. 1B), which appears contradictory to previous studies^4,10^. To understand this result, it is vital to analyze consistent spatial patterns in drought trends. Linear trends for each 0.5 × 0.5° grid cell were calculated using the binary occurrence (presence/absence) of drought within the cell. Drought occurrence was defined in the same manner as for the European scale, i.e. the 20^th^ percentile. In this way, trends in drought occurrence at the grid scale were calculated in an analogous manner to the previous trends in drought area, or the area-weighted sum of binary occurrences across Europe. Trends in drought occurrence were constrained to be linear and presented as the rate of change in drought likelihood (%) per decade, *k*_*SPI*6_ and *k*_*SPEI*6_.

**Figure 2:**
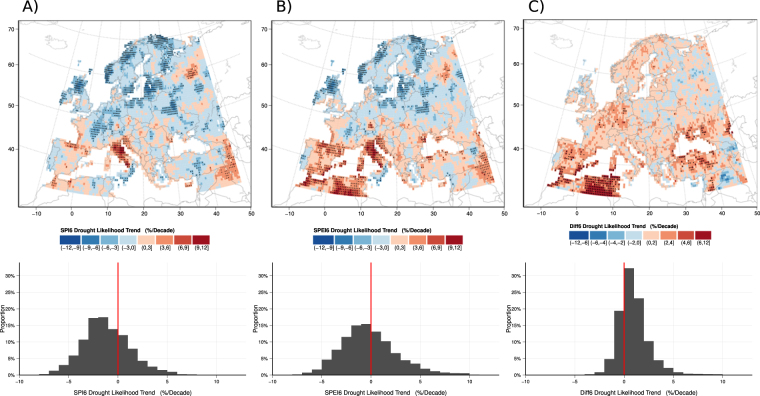
Spatial distribution of (**A**) *k*_*SPI*6_, (**B**) *k*_*SPEI*6_, and (**C**) *k*_*DIFF*6_. Stipples represent statistically significant trends. The distribution of trends for all grid cells is shown below the corresponding map. The color scale for *k*_*DIFF*6_ is modified slightly to match its smaller variance. Figure generated using the ggplot2 package^30^ in R version 3.4.0. (https://cran.r-project.org/).

Spatial patterns in drought occurrence measured by the SPI6 are broadly similar to those measured by the SPEI6, with increasing occurrence across southern Europe and the Mediterranean and decreasing occurrence for much of northern Europe (Fig. 2A and B). In southern Europe and the Mediterranean, drought likelihood has increased at a rate greater than 3% per decade, which is statistically significant based on a t-test of the trend’s slope. For perspective, a 3% increase per decade would increase drought likelihood from 12% in 1958 to 29% in 2014, assuming it passed through the theoretical mean at the reference period mid-point (1985). The most substantial increase in drought frequency occurred in northern Italy, which experienced no detectable SPI6 droughts prior to 1980 followed by frequent, severe droughts throughout the early 1990s and again in 2003 and 2006–07. In contrast, much of northern Europe experienced significantly decreasing drought likelihood since 1958, particularly when measured by the SPI6 (Fig. 2A and B). The most significantly decreasing drought trends occurred in Latvia (−8.35%/decade) and Scotland (−8.60%/decade) for SPI6 and SPEI6, respectively. Some isolated regions do not strictly adhere to the north-south dipole, such as eastern Turkey and northern Russia; however, these regions have been previously identified^24,26,31^ as producing anomalous climate trends, potentially related to low station density, individual gauge issues, or missing data.

The identification of opposing drought trends for northern and southern Europe supports similar findings of a north-south European dipole^10,12,32^. Our results add confidence to these previous findings by applying a more robust regression method that controls for recurrent seasonal patterns and temporal autocorrelation. Notably, our results also closely resemble climate model projections of precipitation that show a drier Mediterranean region and wetter northern Europe^6,33–35^. In this way, our findings place past drought observations firmly into the projected timeline and spatial pattern of European drought impacts due to climate change.

Spatial analysis further highlights the differing trends between the two drought indices and explains why *A*_*SPI*6_ decreased during the study period, in apparent conflict with previous studies. *A*_*SPI*6_ considers Europe as a whole, integrating the opposed increasing and decreasing drought trends by area weighting. So, despite significant increases in drought likelihood for southern Europe, the greater total area in the north outweighs these trends, resulting in an overall decrease in SPI6 drought frequency and no change in SPEI6 frequency. This is further confirmed by comparing the distribution of *k*_*SPI*6_, with a mean trend of −1.13%/decade, to the distribution of *k*_*SPEI*6_, which is centered around zero, despite highly positive trends, shown by positively-skewed distributions of both indices (Fig. 2).

Unlike the north-south dipole of drought trends, the difference, *k*_*DIFF*6_, between trends measured by the two indices (i.e. *k*_*SPEI*6_ − *k*_*SPI*6_) is almost entirely positive across Europe (Fig. 2C). This means that considering ET_0_ increases the relative likelihood of drought by an average of 0.97%/decade (95% range of −1.5 to 5.9%/decade) regardless of location, which is then overlain on the north-south patterns of increasing and decreasing precipitation-based drought. Thus, in regions with a decrease in precipitation-based drought likelihood, such as Germany, inclusion of ET_0_ shifts this trend to become positive. In regions near the Mediterranean that already show an increase in precipitation-based drought likelihood, inclusion of ET_0_ exacerbates the observed trend.

### Trends in Evapotranspiration Components

Because ET_0_ represents the primary difference between the SPI and SPEI indices, it is critical to verify the role of ET_0_ in the resulting drought trends, to test the sensitivity of the results to ET_0_ calculation methods, and to identify the specific climate components that drive the increasing deviation between *A*_*SPEI*6_ and *A*_*SPI*6_. ET_0_ is calculated in this study by the Penman-Montieth equation using the Hargreaves’ simplification for daily radiation^36^. This follows the recommendations given in FAO-56 (ref.^37^, Eq. 50), using diurnal temperature difference (*T*_*max*_ − *T*_*min*_) as a proxy for daily solar radiation, while also considering the direct effects of mean daily temperature and wind speed.

**Figure 3:**
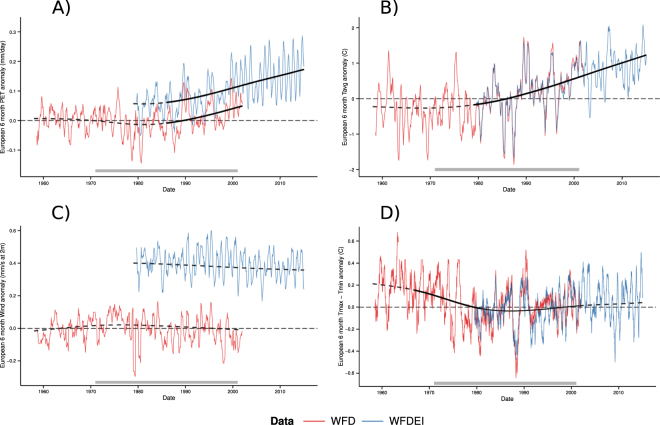
Mean six month seasonal anomaly averaged across Europe for reference evapotranspiration (**A**) and its constituents: (**B**) daily *T*_*Mean*_, (**C**) wind speed, and (**D**) *T*_*Max*_ − *T*_*Min*_. Historical data are shown in red (WFD) and blue (WFDEI). No bias correction is included to highlight differences between WFD and WFDEI. Long term trends are shown as a dotted line, with significant trends as a solid, black overlay. A grey bar at the bottom shows the reference period.

Trends in ET_0_, as well as other constituent climate variables, were calculated using the same non-linear regression techniques as for drought area (Fig. 3. The resulting trend in 6-month European ET_0_ anomaly has a nearly identical shape to *A*_*DIFF*6_ (Fig. 3A), which exhibits a step-change decrease in the late 1970s followed by a decade of low ET_0_ and a continuous, statistically significant increasing trend beginning in the late 1980s that continued until the end of the study period (2014). The observed steady increase in ET_0_ since the late 1980s is supported by other studies that have noted increases in European evapotranspiration during the same time period^38,39^, although our results are unique in providing a trend measure for drought occurrence/frequency. It should be noted that any consistent bias between the WFD and WFDEI was accounted for in the regression model using the term *β*_*Data*_, producing overlapping data and trends as shown in Fig. 1. Trends for the evapotranspiration components also have this correction, producing a continuous and overlapping trend; however, Fig. 3 shows the data and trends without this correction for transparency and to highlight potential differences between the datasets.

**Figure 4:**
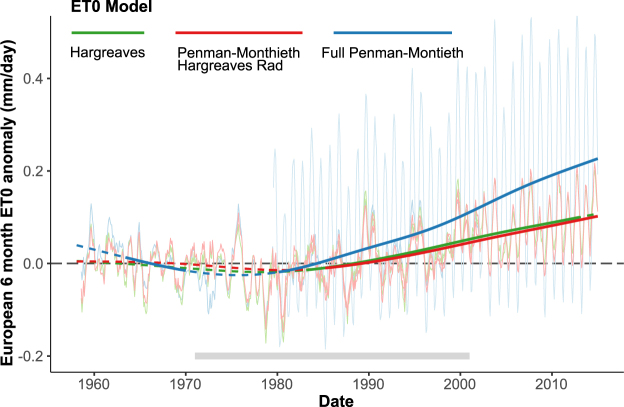
Anomalies in six month mean reference evapotranspiration calculated using three models of increasingly complexity and data requirements. WFD and WFDEI time series are plotted separately in light colors, whereas the spline trend is shown in bold.

Several methods exist to calculate ET_0_; therefore, the choice of method is critical as it may affect observed trends^16^. To test the robustness of the results to the choice of estimation procedure, we compared our ET_0_ trends with those calculated using the more simplistic Hargreaves’ equation (which ignores wind speed) and the more complex FAO-56 version of the Penman-Montieth equation (which adds radiation and humidity terms). These results (Fig. 4) confirm that the ET_0_ trend is consistent in shape across all models and that the method chosen here is conservative with respect to trend magnitude. The results also point to a notable discontinuity between the WFD and WFDEI time series when using the full Penman-Montieth equation, which produces a difference in ET_0_ variance that repeats seasonally (Fig. 4). This discontinuity is due to a previously observed issue in processing downward shortwave radiation to account for monthly aerosol and cloud cover in the WFDEI^26^. Despite this discontinuity, it is important to note that the Penman-Montieth trend using the WFD matches closely all other ET_0_ methods until this dataset ends in 2001. Thus, we chose to use the Penman-Montieth equation with a Hargreaves’ simplified radiation. It is currently the most complex ET_0_ formulation that avoids the discontinuity in radiation and statistical accounting for differing variance, while providing a slightly conservative trend consistent with all other models.

ET_0_ represents the hypothetical atmospheric water demand from a fully wetted surface, but is not strictly equivalent to actual evapotranpiration, which is limited by surface water availability. However, ET_0_ provides a valuable estimate at the continental-scale where actual evapotranspiration is challenging to calculate and has a value of its own, particularly for the fully humid climate of Europe and when values are normalized, as in the SPEI.

The three major climate components of ET_0_ in this study, daily *T*_*mean*_, *T*_*max*_ − *T*_*min*_, and wind speed, each contributes differently to the observed ET_0_ trend and ultimately, to the deviation between SPEI6 and SPI6. Mean daily temperature is the primary driver of increasing ET_0_, showing a significant increasing trend that begins in early 1979 and continues until 2014 (Fig. 3B). During this period, the overall increase in the smoothed six month *T*_*mean*_ anomaly is 1.4 °C, or 0.39 °C per decade, producing the highest *T*_*mean*_ anomaly in 2014 (Fig. 3B). Mean temperature anomalies since 2000 rarely fell below 0 °C, the median value during the reference period, supporting the statistically significant trend towards higher temperatures, and thereby higher reference evapotranspiration, in the latter part of the time series. Such an increase is consistent with observed temperature change across the continent during this time period^24,31,40^ as well as with climate change projections of continental temperature increases^33,35^.

Wind speed and diurnal temperature difference produce secondary effects within the ET_0_ trend. Mean European wind speed has no statistically significant long-term trend (Fig. 3C); but it does explain the consistent bias between the WFD and WFDEI datasets (see Methods section for details regarding controlling for bias in regression). The difference between WFD and WFDEI wind speed has been noted previously^28^ and is a function of methodological differences between the underlying ERA-40 and ERA-Interim climate data. After removing this bias, there is a minor peak in wind speed during the 1970s followed by a slight decrease, which may be related to global stilling^41^; however, the trend is not significant, implying that wind speed most likely plays a minor role in the observed deviation between *A*_*SPEI*6_ and *A*_*SPI*6_. Diurnal temperature difference, *T*_*max*_ − *T*_*min*_, used as a proxy for daily solar radiation, underwent a statistically significant step-change decrease between 1967 and 1980 (Fig. 3D). The 1970s step-change corresponds to a decrease in ET_0_ during the same period, delaying the effect of already increasing mean temperatures and thereby causing a temporary decrease in *A*_*DIFF*6_. The downturn in diurnal temperature range during this period has been noted in other studies^42,43^ and has been linked to a shift from a period of global dimming to a period of global brightening^44^ or to shifts in large scale circulation patterns^45^.

### Implications

This paper reveals, for the first time, an increasing deviation in European regional drought area and frequency measured by two prominent and related drought indices (SPI and SPEI). This divergence is driven primarily by an increase in temperature from 1979 until 2014, which produced a consistent increase in ET_0_, delayed until the late 1980s by the secondary effects of a step-change in diurnal radiation. Both the SPI and SPEI indicate that drought frequency has increased in southern Europe and decreased in northern Europe. However, the inclusion of reference evapotranspiration in the SPEI, driven by a steady increase in European temperature, explains the index divergence, enhanced SPEI droughts in the south, and a northward shift of increased drought frequency over time. A northward shift of water balance drought has important implications for future water management and European agriculture^46^.

This continental-scale disparity in wetting and drying combined with the rapid onset and continuously increasing deviation is highly consistent with both the timeline and spatial pattern of projected climate change impacts for Europe. Further, it is consistent with findings for precipitation and evapotranspiration divergences at a global scale^47^. This suggests that observations and predictions have converged and also supports the claim that climate change has already produced measurable effects on European drought. These exploratory results provide valuable observations of an observable climate change imprint on European drought occurence and will hopefully motivate attribution studies like^48,49^ to focus on the growing divergence between SPI and SPEI-based drought. Detection of this subtle, but critical deviation among two of the most used meteorological drought metrics highlights the challenge and importance of considering drought trends in a non-stationary climate, particularly when communicating changes in drought risk to stakeholders and policy makers.

## Methods

### Drought Indices

SPI^17,18^ and SPEI^19^ were calculated following the method outlined in^50^, using a six month accumulation period and one month time step. A six month accumulation period was selected to model seasonal droughts and to reduce the influence of snowmelt timing. SPI6 and SPEI6 were generated for each 0.5° × 0.5° grid cell individually. Normalization was always relative to the WFD during the reference period 1/1/1971 to 12/31/2000 so that results for the WFD and WFDEI were comparable directly, while also matching the WMO standard for 30-year climate normals. Normalization of the precipitation-based (SPI) and climatic water balance (SPEI) was based on the two-parameter gamma distribution and Generalized Extreme Value (GEV) distribution, respectively, following recommendations in^50^. Drought indices were calculated separately for WFD and WFDEI to avoid artificially merging the climate data sets and to highlight potential differences in findings based on their slightly different underlying atmospheric models.

### Climate Data

All climate data used in this study is based on the Watch Forcing Data (WFD)^25^ and the Watch Forcing Data Era-Interim (WFDEI)^26^, which cover the periods 1/1/1958–12/31/2001 and 1/1/1979–12/31/2014, respectively. These datasets are based on the well-reviewed ERA-40^27^ and ERA-Interim^28^ gridded climate data, respectively, but have undergone spatial interpolation to improve the resolution to 0.5 × 0.5°, while undergoing additional validation against observed climate records^26^ and bias correction based on gauge data. Bias correction is based on CRU monthly data, with the WFD correction based on CRU TS2.1 and the WFDEI based on CRU TS3.1, TS3.101, and TS3.21^51^.

Precipitation is calculated as the sum of rainfall and snowfall, whereas ET_0_ is calculated by the Penman-Montieth Equation using the Hargreaves’ simplification to derive solar radiation from the diurnal difference between maximum and minimum temperature (*T*_*max*_ − *T*_*min*_) (ref.^37^, eq. 50). Calculation of reference evapotranspiration otherwise follows the FAO-56 Penman-Montieth method^37^. It should be noted that reference evapotranspiration is not equivalent to actual evapotranspiration, which is limited by surface water availability. The Hargreaves’ simplification was used to avoid a previously discovered discontinuity between the WFD and WFDEI downward shortwave radiation^26^ that affected the full FAO-56 Penman-Montieth equation (ref.^37^, eq. 6). Sensitivity of the results to the ET_0_ methodology was quantified by comparing ET_0_ calculated using the FAO-56 equation with Hargreaves’ radiation simplification to the more complex, full FAO-56 equation and simpler Hargreaves’ equation^36^. Use of the FAO-56 equation with Hargreaves’ radiation was found to be reasonable and conservative, representing the most complex ET_0_ formulation that also avoids issues with the WFD/WFDEI radiation discrepancy.

### Drought Trend Analysis

Analysis is divided into three steps. First, separate trends in percent European drought area were calculated based on the SPI6, SPEI6, and the difference between their drought area. Then, trends in SPI6 and SPEI6 drought occurrence were determined for each grid cell, allowing for a spatial comparison of trend patterns. Finally, trends in the constituents of SPEI across the European domain were tested to determine the role of each component in explaining the observed differences between SPI6 and SPEI6.

For the purposes of this study, Europe is defined by the domain (−10° to 48°E) by (33° to 72°N). Iceland and the Azores are not included in this definition of Europe. A cell is considered to be in drought if the SPI6 or SPEI6 <−0.84, corresponding to the 20th percentile. Thus, European drought area is the percent area below this threshold. In addition to calculating the percent drought area for SPI6 and SPEI6, referred to as *A*_*SPI*6_ and *A*_*SPEI*6_, respectively, the difference in drought area estimated by these two indices was calculated as

1 $${A}_{DIFF6}={A}_{SPEI6}-{A}_{SPI6}$$

Trends in *A*_*SPI*6_ and *A*_*SPEI*6_ were calculated by using a logit link, ${\rm{logit}}({\pi }_{i})=ln(\frac{{\pi }_{i}}{1-{\pi }_{i}})$, which models the proportion of binary occurrences, *π*_*i*_, between 0 to 100%. Because *A*_*DIFF*6_ is instead bounded by −100% and 100%, trends in *A*_*DIFF*6_ were calculated using a standard Gaussian model. Each trend was fit using the general equation:

2 $${\rm{logit}}({A}_{SPI6})={f}_{Trend}({\rm{Date}})+{f}_{M}({\rm{Month}})+{\beta }_{Data}+{\beta }_{0}+{\varepsilon }_{t}$$

where

3 $${\varepsilon }_{t}=\sum _{n=1}^{n=3}\,{\varphi }_{n}{\varepsilon }_{t-n}+{u}_{t}$$

in which *f*_*Trend*_(*Date*) represents a spline curve response to the calendar date between 1/1/1958 and 12/31/2014, *f*_*M*_(*Month*) is a 12 month cyclic cubic spline constrained to ensure continuity across each new year, *β*_*Data*_ is an intercept that accounts for differences between the WFD and WFDEI, *β*_0_ is the model intercept, and *ε*_*t*_ is a term that can account for temporal autocorrelation among the model errors. Autocorrelation is modeled by an AR term of 1–3 month lags, defined by *ϕ*_*n*_ *ε*_*t*−*n*_ where n is the AR month lag. In this way, *f*_*Trend*_() measures a long-term trend in *A*_*SPI*6_ without imposing a linear requirement, while the remainder of the model accounts for other patterns and factors that could affect the trend term. All non-significant terms were removed from the final model. Regression fitting was performed using the mgcv package in R^52^.

Trends for *A*_*DIFF*6_ were calculated by a similar approach, using a Gaussian model rather than the logit transform:

4 $${A}_{DIFF6}={f}_{Trend}({\rm{Date}})+{f}_{M}({\rm{Month}})+{\beta }_{Data}+{\beta }_{0}+{\varepsilon }_{t}$$

where

5 $${\varepsilon }_{t}=\sum _{n=1}^{n=3}\,{\varphi }_{n}{\varepsilon }_{t-n}+{u}_{t}\quad \quad {u}_{t}=N\mathrm{(0,}\,{\sigma }^{2})$$

Trend significance was calculated by determining whether the instantaneous first derivative of the *f*_*Trend*_() spline term was significantly different from zero using a t-test with *α* = 5%.

Spatial trends were calculated by a similar model, but instead used the binary occurrence of drought at each grid cell rather than the percent area across Europe. The regression model therefore used logistic regression, but assumed a linear trend for each grid cell to allow for easier comparisons of trend slopes:

6 $${\rm{logit}}({\pi }_{SPI\mathrm{6 < }-0.84})={k}_{SPI6}({\rm{Date}})+{f}_{M}({\rm{Month}})+{\beta }_{Data}+{\beta }_{0}+{\varepsilon }_{t}$$

where

7 $${\varepsilon }_{t}=\sum _{n=1}^{n=3}\,{\varphi }_{n}{\varepsilon }_{t-n}+{u}_{t}$$

where *k*_*SPI*6_ is the long-term trend for the SPI6 drought likelihood. This trend in likelihood is presented as a percent change in drought occurrence per decade. Long-term differences between SPI6 and SPEI6 drought trends at the grid cell resolution are calculated by subtracting trends in drought occurrence for each variable:

8 $${k}_{DIFF6}={k}_{SPEI6}-{k}_{SPI6}$$

where *k* is the trend in drought likelihood and subscripts refer to the drought index or the difference between their likelihoods. Statistical significance was calculated for *k*_*SPI*6_ and *k*_*SPEI*6_ using a t-test of the trend’s slope, while the statistical significance of *k*_*DIFF*6_ was calculated following^53^.

Trends in the constituent climate variables were determined by first calculating the 6-month seasonal anomaly for each variable: ET_0_, *T*_*mean*_, *T*_*max*_, *T*_*min*_, *T*_*max*_ − *T*_*min*_, and 2 m wind speed. This involved calculating the six month moving average for each constituent at each grid cell and subtracting the seasonal mean using the same reference period as defined for the SPI and SPEI. The seasonal anomaly for Europe was then calculated as the area-weighted mean of these anomalies. In this way, seasonal anomalies are handled in exactly the same manner as SPI and SPEI, making them comparable. Trends were then calculated using the same Gaussian models described in Equations 4 and 5.
